# Arsenic as Traditional Chinese Medicine Provides New Hope for Overcoming High Treatment Costs of Acute Promyelocytic Leukemia

**DOI:** 10.1200/JGO.2016.005405

**Published:** 2016-07-06

**Authors:** Hong-Hu Zhu, Jiong Hu, Xue-Fei Gu

**Affiliations:** **Hong-Hu Zhu**, Peking University People’s Hospital, Beijing, China; **Jiong Hu**, Shanghai Jiao Tong University School of Medicine, Shanghai, China; **Xue-Fei Gu**, China National Health Development Research Center, Beijing, China

The use of targeted treatment and chemotherapy has rendered acute promyelocytic leukemia (APL) a highly curable form of cancer. A landmark study showed that APL can be cured without chemotherapy using arsenic trioxide (ATO) and all-*trans*-retinoic acid (ATRA).^1^ The former can even be substituted with oral arsenic (the Realgar-*Indigo naturalis* formula), allowing a largely home-based treatment protocol.^2^ The combined use of ATO and ATRA as a first-line treatment of APL has been adopted by the most recent National Comprehensive Cancer Network guidelines.^3^

**Fig 1:**
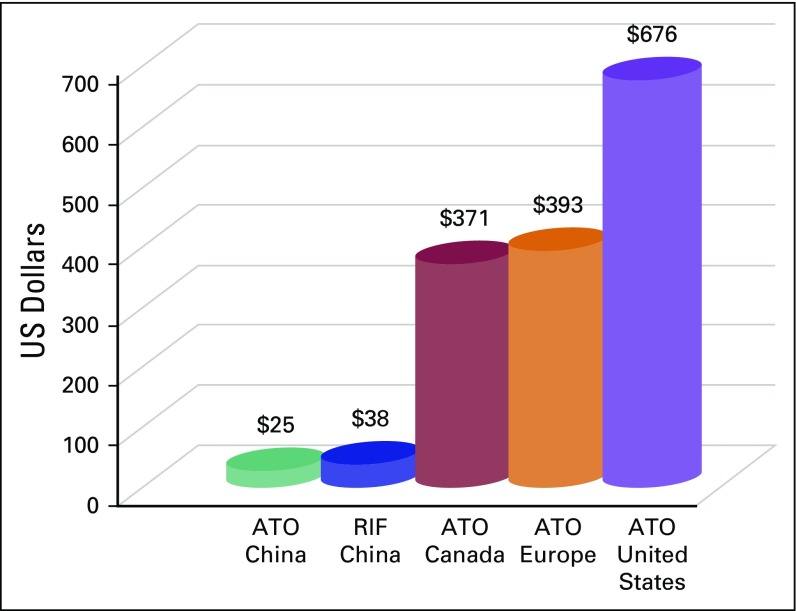
The median costs of intravenous arsenic trioxide (ATO) or oral arsenic (the Realgar-*Indigo naturalis* formula [RIF]) per day in China and other countries.

However, the high price of ATO in Western countries is a challenge to the health care system, limiting the maximization of its benefits. The price of ATO is approximately $676 in the United States, 530 Canadian dollars in Canada, and €393 in Europe (Fig 1). Three-year cumulative pharmacy costs for ATO plus ATRA were higher than ATRA plus chemotherapy (€46,700 *v* €6,500 per patient) from an Italian payer perspective.^4^ However, from a Canadian perspective, a combined ATO plus ATRA treatment is associated with incremental cost-effectiveness ratios of 50,193 Canadian dollars per quality-adjusted life-years.^5^ Moreover, in the United States, compared with the ATRA plus idarubicin regimen, first-line ATO plus ATRA treatment had incremental cost-effectiveness ratios of $4,512 per life-year saved and $5,614 per quality-adjusted life-year gained.^6^ These figures have to be put in context with the fact that 70% of countries in the world, which contribute to > 75% of the world’s population, have a gross national income per capita of < $10,000. Therefore, reducing the price of ATO and adopting an outpatient treatment model may overcome this problem.

China has taken important measures to improve the outcomes of patients with APL. First, the price of 10 mg of ATO is approximately $25 (¥160) and that of 10 mg of ATRA is roughly $0.2 (¥1.5) under the government price regulation policy. The total pharmacy cost of ATO and ATRA treatment is approximately $3,626 after the completion of an entire round of treatment using combined ATO and ATRA as a first-line treatment of non–high-risk patients with APL (accounting for roughly 80% of all cases of APL). The gross domestic product per capita of China was $7,603 in 2014, which makes the costs of the two drugs affordable even by out-of-pocket payment standards.

Second, 70% to 85% of the costs of the two drugs are covered by three health insurance schemes (Basic Insurance for Urban Employees, Basic Insurance for Urban Residents, and the New Rural Cooperative Medical Scheme), which cover > 95% of the population. Therefore, the direct economic burden of patients with APL is significantly reduced. Third, we propose a novel oral arsenic and ATRA–based outpatient treatment model to reduce the medical costs. We previously found that oral arsenic (Realgar-*Indigo naturalis* formula) provided a similar outcome compared with intravenous ATO using a chemotherapy-included protocol in patients with APL^7^ and showed a reduced cost in the oral arsenic group (median, $13,183.49 *v* $24,136.98; *P* < .001).^8^

We recently reported excellent, albeit preliminary, outcomes in 20 patients with non–high-risk APL treated with oral arsenic and ATRA via a largely home-based treatment protocol.^2^ Impressively, the median of the total medical costs was $4,675 (range, $3,174 to $12,698). Patients resumed their usual lifestyles during postremission therapy, and their quality of life was rated as nearly normal. A prospective, multicenter, randomized trial is underway in China (Chinese Clinical Trial Registry No. ChiCTR-TRC-13004054), which has completed recruitment; preliminary results are awaited.

Importantly, APL is a model of how targeted therapies on their own can trigger definitive cures and show the power of traditional medicine. This Chinese model of APL treatment is worth promoting, especially in undeveloped countries. With its low costs and the merits of oral administration, traditional medicine may bring hope to overcoming the high cost of the treatment of cancer.
